# Expanding the reach of a fall prevention intervention for older adult emergency department patients through telehealth: a trial protocol

**DOI:** 10.3389/fpubh.2025.1720938

**Published:** 2025-12-17

**Authors:** Audrey Keleman, Megan Bounds, William Mundo, Jonathan Gomez-Picazo, Meredith Mealer, Sunny A. Linnebur, Bucky Ferozan, Julie Dunn, Jennifer Stevens-Lapsley, Elizabeth Goldberg

**Affiliations:** 1 Department of Physical Medicine and Rehabilitation, University of Colorado Anschutz, Aurora, CO, United States; 2 Eastern Colorado Geriatric Research, Education, and Clinical Center, Department of Veterans Affairs, Aurora, CO, United States; 3 Department of Emergency Medicine, University of Colorado Anschutz, Aurora, CO, United States; 4 Department of Clinical Pharmacy, University of Colorado Anschutz, Aurora, CO, United States; 5 Emergency Medicine, UCHealth Medical Center of the Rockies, Loveland, CO, United States; 6 Trauma and Acute Care Surgery, UCHealth Medical Center of the Rockies, Loveland, CO, United States

**Keywords:** falls, fall prevention, adaptations, telehealth, remote, emergency department, rural

## Abstract

**Background:**

Falls are a leading cause of emergency department (ED) visits among older adults, yet many patients are discharged without a clear understanding of their fall risk factors or access to timely prevention services. The Geriatric Acute and Post-Acute Fall Prevention (GAPcare) intervention has demonstrated efficacy in reducing fall-related ED revisits by addressing these factors during the ED visit. Still, its reliance on in-person specialists limits scalability to smaller and lower-resourced EDs.

**Methods:**

This study outlines a protocol for adapting GAPcare into a telehealth-enabled model (e-GAPcare) using a two-phase implementation science approach. Phase 1 engages ED staff, patients, and caregivers in structured workgroups to guide the adaptation of telehealth while preserving core intervention components. Phase 2 involves a single-arm trial at one ED with 40 older adults presenting after a fall, evaluating feasibility and acceptability. The intervention includes remote consultations with a pharmacist and physical therapist, tailored assessments to address individual fall risk factors, and a standardized checklist integrated into discharge planning that is shared with primary care clinicians.

**Outcomes:**

Data will be collected through surveys and electronic health records over a 6-month follow-up period. Trial outcomes include usability, care transition quality, healthcare utilization, and uptake of recommendations.

**Conclusion:**

By leveraging hospital-based telehealth infrastructure and eliciting end-user perspectives on needed adaptations, e-GAPcare aims to extend the reach of evidence-based fall prevention programs to smaller EDs or those that lack in-person pharmacists and physical therapists.

## 1 Introduction

Falls are the leading cause of injury-related emergency department (ED) visits among older adults (1). Community-dwelling older adults who visit the ED after a fall have a 30% increased risk of falling again within 6 months compared to age-matched peers, and experience greater declines in functional abilities such as balance after ED discharge (2). Even though ED clinicians routinely screen for fall risk, many older ED patients leave the ED without an understanding of why they fell and how to reduce their fall risk (3, 4). Those patients who are referred to outpatient physical therapy or other prevention resources often fail to follow-up or experience delays in receiving these services, resulting in recurrent falls in the high fall risk period immediately after ED discharge (4, 5). The Geriatric Acute and Post-Acute Fall Prevention (GAPcare) intervention bridges this gap by providing fall prevention services in the ED. GAPcare had high patient and caregiver satisfaction (6–8), did not prolong ED length of stay (8), and resulted in 66% fewer 6-month fall-related ED visits compared to usual ED care (9).

The original GAPcare approach limits scalability to large EDs that have in-person pharmacist and PT coverage. Large EDs account for less than half of all ED visits (10); the original approach misses fall prevention opportunities for 3 million older adults who seek care for falls in smaller EDs each year (11). In this study, we adapt the GAPcare intervention to be implemented via telehealth (e-GAPcare) in a smaller ED context, which will expand the reach of the intervention to communities that lack the staff and resources to initiate GAPcare as currently designed. e-GAPcare leverages hospital-based internet and does not require users to have broadband, Wi-Fi, or devices. Implementation of telehealth in smaller EDs has been associated with 30% lower annual ED costs (12) and improvement in access to care (13). Telerehabilitation conducted by PTs (14–16) and telepharmacy (17–20) consultations are feasible, but require further testing among older patients in the ED setting (21).

This paper describes a protocol for adapting GAPcare to be implemented in a smaller, lower-resourced ED via telehealth while maintaining its essential core components. In Phase 1, we will elicit perspectives from content experts in telehealth, ED staff, and patients using study-facilitated workgroups to inform the practical considerations of initiating a telehealth intervention, including software, devices, institutional agreements, funding, and billing of services. In Phase 2, we will determine the acceptability and feasibility of e-GAPcare by conducting a single arm study with community-dwelling older ED patients (*n* = 40) with a recent fall.

## 2 Materials and methods

### 2.1 Phase 1

#### 2.1.1 Creation and function of the workgroup

Using purposive sampling, we will form a workgroup comprising ED personnel and patients/caregivers to actively participate in all stages of adaptation. Purposive sampling will be guided by demographic and clinical characteristics that reflect the broader ED population impacted by fall risk and ED care transitions. ED personnel will be invited to join based on their roles, and patients/caregivers will be solicited by clinicians from the community associated with the hospital. ED patients and caregivers (*n* = 5) will work with technicians, registered nurses, advanced practice providers, and physicians (*n* = 5), administrators (*n* = 5), and pharmacists and PTs (*n* = 5).

#### 2.1.2 Conceptual model and guiding principles

Workgroup members will be guided to think of ways to improve the fit with the smaller ED context without changing core elements of GAPcare that are deemed necessary for efficacy. Core elements include initiating GAPcare in the ED, number and length of program sessions, pharmacist and PT consultations, automated communication with outpatient clinicians, and use of motivational interviewing. We will focus the discussion and refinement of the e-GAPcare prototype on four elements important to the patient experience, “post-COVID,” as promulgated by Boissy as part of the Maturity Model for Patient Experience (22): teamwork, empathy, safety, and ease. By focusing on these four elements, we will gather feedback on making the e-GAPcare site specific and person-centered. Questions and suggested measures for successful completion of the study in Phase 2 are listed in Table 1. The workgroup will modify these measures depending on site, staff, patient, and caregiver priorities (see Supplementary material). In each workgroup, participants will become familiar with the GAPcare intervention and proposed adaptations to deliver remotely. The first two workgroups will reach consensus on these adaptations and the third will pilot the protocol with a mock patient. The full workgroup agendas are in Supplementary material. We will track and report all adaptations using the template for intervention description and replication (TIDieR) checklist and guide (23), in addition to the Model for Adaptation Design and Impact (MADI) (24).

**Table 1:** Workgroup guiding principles, derived from the maturity model for patient experience (22).

Category	Questions	Measures for feasibility study in Phase 2 (to be modified by workgroup)
Teamwork	How would you like to see the healthcare team work together? (staff) How would you like to be involved in your care? (patient/caregiver)	% report GAPcare team worked together as a team to care for them % report being included/engaged in their care
Empathy	How can we improve our plans to ensure care is provided in a way that respects patient/staff preferences/needs?	% patients report improved care transitions with intervention (CTM-3) (49) % patients with social determinants of health addressed proactively % who would recommend e-GAPcare to a friend (Net Promoter score)
Safety	What safety concerns do you have regarding the use of telehealth? What is staff experience and education regarding safe mobility in the ED?	Number of adverse events per participant Number of safety event reporting system events
Ease	What frustrates you about using technology for health applications? What would the ideal telehealth intervention look like?	% timely assessments (<30-min time-to-consult-initiation) % ease of tech [System Usability Scale (50) score and Health Information Technology Usability Evaluation Scale (51) score] % connectivity issues, early abortions, conversions to call only

#### 2.1.3 Analysis plan (qualitative)

We will review workgroup meeting transcripts, independently code the data, and produce a list of themes and subthemes following a rapid analytic approach (25), a form of directed content analysis (26). We will start with a calibration process and jointly review a subset of transcripts to ensure consistency in applying codes before moving to independent coding. We will iteratively search for common themes and subthemes across participants and interviews that are patterns across participant responses. We will review themes in relation to the coded extracts and the entire dataset, and select representative quotes from the interviews to illustrate these themes. Coding definitions and decisions as well as ideas about emerging themes, will be recorded in an ongoing audit trail (27). We will prepare the analytic narrative and contextualize it using the existing literature. We will use framework analysis – a rapid qualitative analysis technique in which investigators summarize content within categories into charts after transcription (28, 29) – as it is particularly well-suited for generating recommendations within a limited time period. The framework analysis will inform Phase 2. We will have an external auditor not part of the qualitative process review the analytic decisions.

### 2.2 Phase 2

Upon completion of Phase 1, we will launch a single-arm trial to evaluate feasibility and acceptability among 40 patients presenting to the ED within 7 days of a fall. All participants will receive the e-GAPcare intervention (9). Our primary outcome is the feasibility of implementation via telehealth, and secondary outcomes are acceptability, recurrent fall-related ED visits, and other healthcare utilization (see Section 2.2.3 Measures). We will follow participants for 6 months via survey and electronic health record (EHR) data.

#### 2.2.1 Participant recruitment and setting

We will recruit study-eligible patients from the Medical Center of the Rockies ED, a small, non-profit community hospital (36,000 annual ED visits) that serves a disproportionate share of geriatric trauma patients and has a largely rural catchment area. We will assess a consecutive sample of patients presenting to the ED during telehealth on call hours (typically 7:00 a.m. – 7:00 p.m.) for eligibility. We will offer proxy consent to participants who wish to participate but who lack capacity. We assess capacity according to the Colorado Multiple Institutional Review Board procedures which includes asking the patient why the study is being done, what the risks and benefit are, and what are the benefits of the study. Participants will have three opportunities to answer questions correctly. Study participation will occur after written informed consent is obtained from the patient or their proxy (for those lacking capacity). All patients will be screened for dementia with the AD8 Dementia Screening Interview (30). It is estimated that 40% of older ED patients have dementia (31, 32), but few are diagnosed. For those who newly screen positive, scoring two or greater on the AD8, we will offer resources to the patient for further testing and information.

Characteristics of patients eligible for the study include: community-dwelling adult (non-institutionalized) 65 years-old or older presenting to ED after a fall [accidental fall; fall not due to syncope or external force (i.e., struck by car or assault) and fall not due to serious illness (i.e., stroke, acute myocardial infarction)], planned discharge to home/assisted living/rehabilitation at completion of ED visit (i.e., not admitted), and presence of a proxy to give informed consent if patient lacks capacity. Exclusion criteria consist of inability to provide informed consent due to intoxication or acute change in mental status, or presence of injuries that prevent mobilization (i.e., pelvic or lower extremity fractures).

#### 2.2.2 Study procedures

**Figure 1:**
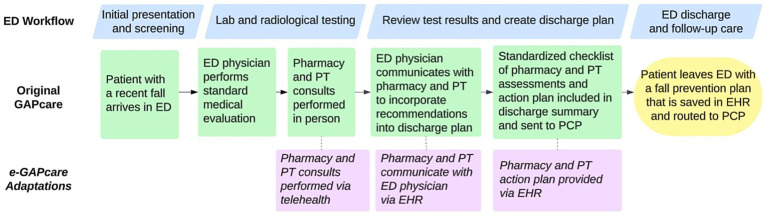
Flow through of patient experience in e-GAPcare. The figure displays steps of GAPcare within the context of a typical ED workflow and highlights the adaptations for e-GAPcare. ED, emergency department; PT, physical therapy; EHR, electronic health record; PCP, primary care physician.

Figure 1 displays the process of e-GAPcare from a patient perspective and highlights adaptations made from the original intervention. Table 2 further describes planned implementation adaptations.

**Table 2:** Planned Implementation adaptations for e-GAPcare (MADI) (24), to be refined and completed by workgroup and research team in Phase 1.

Adaptation characteristics for e-GAPcare	Possible mediating or moderating factors	Implementation and intervention outcomes (intended and unintended)
Device connected to hospital WiFi, brightness and contrast increased, text and icon size set to maximum, extraneous content reduced, cleaned with sanitizing wipes between uses	Alignment with target population by simplifying functions and making intended use more intuitive	Acceptability Appropriateness
Screen mounted on a mobile cart	Alignment with target population by simplifying functions and making intended use more intuitive, and fit within existing clinical workflow (easily mobile)	Feasibility
One page user guide for device provided and in-person demonstration	Alignment with target population by simplifying functions and making intended use more intuitive	Feasibility
Basic device competencies assessment	Assess whether the telehealth assessment and device setup will work for the patient	Appropriateness
If basic device competencies are not met, caregivers or research staff to assist	Align with clinical workflow as needed	Appropriateness
Local staff to be trained in safe mobility while patient is performing telehealth assessments (such as gait belt use and providing support)	Required for safety of evaluations conducted over telehealth	Appropriateness

##### 2.2.2.1 Telehealth procedures

We will leverage the ED’s existing devices for the consultations which include iPads mounted on a mobile cart. The screen is plastic encased and connects to the hospital’s Wi-Fi server. We will take several steps to optimize the device for older adults: we will increase the brightness and contrast, maximize text and icon size, and reduce extraneous content. We will clean iPads with sanitizing wipes between uses. Demographic, baseline cognition, mobility, and fall occurrence data will be collected into the HIPAA-compliant REDCap program by the research staff. Video consultations with pharmacists and PTs will take place using a video-conferencing platform identified as user-friendly and hospital approved in Phase 1 (see Table 2 and Figure 1).

###### 2.2.2.1.1 Training and field testing

Each participant will receive a screen, an illustrated one-page step-by-step user guide, and an in-person demonstration of the device and video conferencing platform. Research staff will ask patients to perform basic device competencies (e.g., orient the device toward face, turn it on, open the video conferencing app) under the direct supervision of the research staff in the ED. If the participant cannot demonstrate basic use, they will be assisted by caregivers or the research staff, depending on their preferences. Research staff will complete a checklist of items successfully completed by each patient, along with the timing of completion, for our feasibility outcome. The camera will be set up to provide the telehealth consultant with a full view of the exam room. Research staff will ensure the camera and microphones are working correctly and position the camera, as necessary.

##### 2.2.2.2 ED clinician care

The ED clinician will perform a standard medical evaluation. Thereafter, the research staff will page the pharmacist and PT on a telehealth call. After the telehealth components, the pharmacist and PT will communicate with the ED clinician via secure chat and post their consults into the EHR. The ED clinician will develop the action plan and incorporate pharmacy and PT recommendations into the discharge paperwork.

##### 2.2.2.3 E-GAPcare pharmacy consult

Pharmacists will evaluate the patient over video. They will (1) perform a medication review using the American Geriatrics Society Beers criteria (33) and the Centers for Disease Control and Prevention’s (CDC’s) Stopping Elderly Accidents, Deaths & Injuries Instrument (STEADI) (34), (2) recommend cessation or tapering of medication that increase fall risk using motivational interviewing (medication therapy management) (35), and (3) communicate the medication-related action plan in writing to the patient and ED treatment team.

##### 2.2.2.4 E-GAPcare PT consult

PTs will evaluate the patient over video, with research staff assisting on site. PTs will perform validated screens of mobility and balance following the CDC STEADI instrument as a framework (34). PTs will direct (1) integrative mobility training and lower extremity strength training, (2) make suggestions on the safety of discharge, (3) recommend outpatient services, such as referral to home or outpatient PT, occupational therapy, or discharge to a skilled nursing facility, and (4) communicate the PT action plan in writing to the patient and the ED treatment team. Specific assessments and treatments will be tailored to the patient, as tailoring is beneficial for older adults with varied levels of need (36, 37).

##### 2.2.2.5 Standardized checklist

Participants will return home with a standardized checklist (8) with details of their assessment and action plan. This checklist is based on the STEADI instrument and addresses the patient’s personal risk factors for falls and pharmacist and PT recommendations. Further actions may include recommendations on assistive device use, a plan for a home safety evaluation, PT follow-up, and the need to see other specialists to treat foot problems or vision impairment. This checklist is sent to the primary care physician (PCP) and saved as a consultation note in the EHR for ED staff and other specialists involved in the patient’s care to review.

Our process of integrating STEADI tools was informed by prior work identifying barriers to use of STEADI in clinical contexts (38, 39). First, we have adapted the screening and referral protocol for efficiency of workflow. One of the first step fall risk screens in STEADI, “Three Key Questions,” is automatically a yes because participants have fallen within the past year (inclusion criteria is they are being seen in the ED for a fall) so the referrals to PT and pharmacy do not need to wait for another screener. Second, we will develop EHR tools and modify the EHR prior to implementation with a focus on ease of use and fit within the workflow. We will ensure the workflow clearly delineates roles and responsibilities by provider, and we will establish infrastructure for disseminating information (consultation notes and recommendations checklist are within EHR and are sent to other providers including PCP).

#### 2.2.3 Measures

During the ED visit, research staff will complete the baseline assessment with participants (see Table 3). Assessments may be modified based on feedback from the workgroup in Phase 1. Patient factors (e.g., sociodemographic questions, past medical history, cognition, function) and e-GAPcare implementation factors (e.g., usability, acceptability) will be assessed during the ED visit.

Data collected during follow-up will come from two sources: (1) self-reported or caregiver-reported falls confirmed during regular telephone follow-up, and (2) EHR review.

**Table 3:** Overview of self-reported assessments and timing.

Domain	Construct	Assessment	Baseline	1 Month follow up	3 Month follow up	6 Month follow up
			During ED visit	Telephone follow-up and EHR review		
Patient factors	Sociodemographic	Standard questions	x			
	Past medical history	Medical history, fall in last three months	x			
	Cognition	AD8 Dementia Screening Interview (30)	x			x
	Function	Barthel ADL Index (52)	x			x
e-GAPcare implementation factors	Usability	System Usability Scale (50), Health Information Technology Usability Evaluation Scale (51)	x			
	Acceptability	Net Promoter Score (53)	x			
	Acceptability	Uptake of medication and PT recommendations				x
Outcomes	Care transition quality	Three Item Care Transition Measure (CTM-3) (49)		x		
	Falls and healthcare utilization	Fall occurrences, ED visits, hospitalizations, and reasons	x	x	x	x

##### 2.2.3.1 Self-reported falls and uptake of recommendations

Research staff will call participants at 1, 3, and 6 months to assess subsequent falls, resulting injuries, healthcare utilization, and uptake of recommendations made by pharmacists and PTs.

##### 2.2.3.2 EHR data for falls and healthcare utilization

We will also gather EHR data on subsequent falls, ED visits, and hospitalizations. To increase data collection accuracy (40), we will follow recommendations for manual EHR review and data extraction: (1) create a standardized data extraction form, (2) implement a standardized protocol for data extraction, (3) train the data extractors, and (4) quality assurance by review of a 10% sample. Although patients may visit another hospital, the UCHealth system spans 12 acute-care hospitals and 25 free-standing EDs, all of which use the same EHR. Thus, we anticipate having access to most follow-up data.

#### 2.2.4 Sample size estimation

e-GAPcare is not designed or powered to determine efficacy. However, we need an adequate sample to explore the acceptability and feasibility of our intervention and to help inform sample and effect size estimates for a subsequent randomized controlled trial. Using a negative binomial comparison assuming effect sizes from the original GAPcare trial, with an alpha of 0.05 and power of 0.8, we would need a sample size of 60 to achieve these aims. We will recruit 40 participants and follow them for 6 months. This sample size is based on practical considerations, including budgetary constraints, participant flow, and the number required to evaluate study goals (feasibility and study scope) (41, 42).

#### 2.2.5 Statistical analysis

##### 2.2.5.1 Implementation

We will use descriptive statistics for key parameters of implementation for this study: (43, 44) number of patients screened, eligible, and recruited (Reach); time required to recruit and time to consult (Implementation), number of patients with refusal and retention at each follow-up (Reach), how many participants could use the setup without help and/or what level of assistance was needed (Feasibility). We will track lessons learned during implementation and use them directly to improve our approaches iteratively. We will use descriptive statistics to assess parameters of acceptability: did participants accept pharmacy/PT consults (Reach), did patients and PCPs receive pharmacy and PT action plans (Adoption and Implementation), were there any technology glitches (Implementation), and was there uptake of recommendations by participants (Reach).

##### 2.2.5.2 Healthcare utilization measures

We will collect the rate of falls, as well as the median/mean number of recurrent falls (45), ED visits, and hospital admissions over 6 months. Dates and times for these outcomes will be obtained from telephone assessments and EHR review (9, 46).

## 3 Discussion

Falls among older adults are a persistent and costly public health challenge, particularly in ED settings where patients often receive limited follow-up care (47). Despite routine risk screening, many older adults are discharged without a clear understanding of their fall risk or access to timely prevention services, contributing to high rates of recurrent falls and healthcare utilization (5, 48). The GAPcare intervention reduces fall-related recurrent ED visits but its reliance on in-person PT and pharmacy clinicians restricts its implementation to large, well-resourced EDs. This study addresses a critical gap by adapting GAPcare into a telehealth-enabled model designed to expand access to fall prevention services in smaller and rural EDs.

We have learned several lessons that may be useful for scaling the intervention. (1) If clinicians are not currently licensed at the facility, they will need to go through the medical staff office to obtain licensing to allow them to interact with ED patients. (2) Operational leaders initiating similar interventions may need permission to use existing that may have been purchased by other groups, such as translation vendors or behavioral health services, who may have invested in the devices to use them for their own services (3) Some health systems may be able to bill for telehealth services, but others include it under the facility fee or ED consultation fee, so billing considerations need to be worked out at each individual site. (4) Hardware may need to be retrofitted for use, especially in PT consultations when a wide view is required for physical function assessments. Retrofitting tablets such as with a fisheye lens requires engineering and 3D printing expertise. As with any intervention that impacts ED workflow and the environment, bringing key partners in to discuss the aspects of the intervention that interface with others early can save intervention champions from needing to delay intervention start.

The study design incorporates several strengths. First, it uses a structured implementation science framework to guide adaptations, ensuring that core components of the intervention are preserved while tailoring delivery to a new context. Second, diverse partners are included in the adaptation process, such as ED clinicians, patients, and caregivers. Including these partners enhances the relevance and acceptability of e-GAPcare. Third, the use of qualitative and quantitative methods affords a more comprehensive evaluation of feasibility and acceptability. Lastly, leveraging existing hospital infrastructure for telehealth delivery minimizes barriers related to technology access and supports scalability.

Limitations of the study include its modest sample size and single-arm trial design. While the study size and design are appropriate for testing our recruitment methods and the feasibility of the intervention, a larger study may better capture the full range of diverse perspectives, particularly from individuals presenting to EDs in even smaller, more remote settings. While a randomized design reduces the risk of unmeasured confounders and the ability to make causal claims, a single-arm trial will allow us to effectively test study procedures and gather valuable preliminary data on the implementation of our remote intervention in a smaller ED.

At the end of this study, we will have technology and strategies that are ready for deployment and will inform the methodology of a subsequent multi-site, fully powered RCT examining the efficacy of e-GAPcare in a sample of rural, suburban, and urban communities. e-GAPcare represents a promising and scalable approach to delivering fall prevention services in EDs that lack access to in-person pharmacy and PT clinicians. By leveraging implementation science and telehealth delivery methods, this study provides a foundation for expanding evidence-based interventions to lesser resourced EDs.

## Data Availability

The raw data supporting the conclusions of this article will be made available by the authors, without undue reservation.
