# Supplementary material for: Expanding the reach of a fall prevention intervention for older adult emergency department patients through telehealth: a trial protocol
Source: Front Public Health. 2025 Dec 17;13:1720938. doi: 10.3389/fpubh.2025.1720938 (PMC12753965; doi:10.3389/fpubh.2025.1720938)
Supplement: Supplementary file 1 [file Table_1.docx]

**eGAPcare**

Our over-arching goal is to expand the reach of GAPcare by testing video telehealth GAPcare (**e-GAPcare**) intervention in a community ED without existing pharmacy and PT services. Participants will meet virtually with pharmacists and PTs while in the ED to perform fall risk assessment and create action plans to reduce falls. Action plans are shared with patients, caregivers, and primary care clinicians automatically through the existing electronic health record (EHR) structure.

**Aim 2 Workgroup Material:**

For each workout group meeting will begin by facilitation of the following steps:

1) familiarization with eGAPcare and its core elements

2) reach consensus on adaptations to improve context fit

3) plan piloting and measures

4) pilot

5) report on adaptations.

Next, we will focus the discussion and refinement of the eGAPcare prototype on four elements important to the patient experience “post-COVID” promulgated by Boissy as part of the Maturity Model for Patient Experience: teamwork, empathy, safety, and ease.

**Expected outcome**: The eGAPcare protocol is ready for piloting, is acceptable to clinicians, staff members, patients, and caregivers. The research team has gathered the information needed to be ready to recruit patients.

**1^st^ 90-minute meeting:**

This will consist of clinical staff only; nurses, clinicians, PTs, pharmacists, administrators (n=15)

**Agenda**

1. Familiarization with GAPcare and its core elements using the eGAPcare flyer **(10 minutes)**
2. What specific adaptations may be desired/necessary at MCR to integrate eGAPcare into the workflow? Share barriers/facilitators/potential solutions. **(15 minutes)**
3. Answer any questions **(10 minutes)**
4. Ask participants to split into 3 break-out groups
   1. Discussion aims for each work group and example questions **(15 minutes)**
      1. Teamwork: How would you like to see the healthcare team work together? The intervention requires teamwork between clinicians, nurses, PTs, pharmacists, and potential case managers.
         1. Suggested measures:
            1. % report GAPcare team worked together as a team to care for them
            2. % report being included/engaged in their care
      2. Empathy: How can we improve our plans to ensure care is provided in a way that respects patient/staff preferences/needs?
         1. Suggested measures:
            1. % patients report improved care transitions with intervention (CTM-3)
            2. % patients with social determinants of health addressed proactively
            3. % who would recommend e-GAPcare to friend(s) (Net Promoter score)
5. Bring everyone back together and have each group present conclusions **(15 minutes, 5 minutes per group)**
6. Reach consensus on adaptations to improve context fit**(10 minutes)**
7. Plan piloting measures **(15 minutes)**

**2^nd^ 90-minute meeting:**

Workgroup will consist of the original 15 members plus patients and caregivers (n=5)

**Agenda**

1. Familiarization with GAPcare and its core elements using the eGAPcare flyer **(5 minutes)**
2. Answer any questions **(10 minutes)**
3. Orient personnel on devices and trainings (5 minutes)
4. Ask participants to split into 3 break-out groups
   1. Discussion aims for each work group and example questions **(35 minutes)**
      1. Safety: What safety concerns do you have regarding using telehealth? What is staff experience and education regarding safe mobility in the ED?
         1. Suggested Measures
            1. Number of adverse events per participant
            2. Number of safety event reporting system events
      2. Ease: What frustrates you about using technology for health applications? What would the ideal telehealth intervention look like?
         1. Suggested measures:
            1. % timely assessments (<30-minute time-to-consult-initiation)
            2. % ease of tech (system usability scale^37^ score)
            3. % connectivity issues, early abortions, conversions to call only
5. Bring everyone back together and have each group present conclusions **(15 minutes, 5 minutes per group)**
6. Reach consensus on adaptations to improve context fit **(10 minutes)**
7. Finalize piloting and measures **(15 minutes)**

**3^rd^ 90-minute meeting:**

Workgroup will be same 20 individuals from 2^nd^ meeting

**Agenda**

1. Familiarization with GAPcare and its core elements using the eGAPcare flyer **(5 minutes)**
2. Answer any questions **(5 minutes)**
3. Pilot: run through with mock participant **(30 minutes)**
4. Ask participants to split into 3 break-out groups **(15 minutes)**
   1. Discussion centered on what went right and what could/should be changed
5. Bring everyone back together and have each group present conclusions **(15 minutes, 5 minutes per group)**
6. Reach consensus on adaptations to improve context fit **(10 minutes)**
7. Finalize piloting and measures **(10 minutes)**
